# Age-correlated changes in the canine oral microbiome

**DOI:** 10.3389/fmicb.2024.1426691

**Published:** 2024-07-16

**Authors:** Gregory Kislik, Lin Zhou, Liudmilla Rubbi, Matteo Pellegrini

**Affiliations:** Molecular Cell and Developmental Biology, University of California, Los Angeles (UCLA), Los Angeles, CA, United States

**Keywords:** whole genome sequencing, canine, microbiome, metagenomics, ElasticNet regression

## Abstract

**Introduction:**

Canine oral disease has been associated with significant changes in the oral microbiome rather than the presence or absence of individual species. In addition, most studies focus on a single age group of canines and as of yet, the relationship between canine microbiomes and age is poorly understood.

**Methods:**

This study used a shotgun whole gene sequencing approach in tandem with the Aladdin Bioinformatics platform to profile the microbiomes of 96 companion dogs, with the sourmash-zymo reference database being used to perform taxonomic profiling.

**Results:**

Findings showed significant age correlations among 19 species, including positive correlations among several *Porphyromonas* species and a negative correlation with *C. steedae*. Although a significant correlation was found between predicted and actual ages, ElasticNet Regression was unable to successfully predict the ages of younger canines based on their microbiome composition. Both microbiome samples and microbial species were successfully clustered by age group or age correlation, showing that the age-microbiome relationship survives dimensionality reduction. Three distinct clusters of microbial species were found, which were characterized by *Porphyromonas*, *Conchiformibius*, and *Prevotella* genera, respectively.

**Discussion:**

Findings showed that the microbiomes of older dogs resembled those that previous literature attributed to dogs with periodontal disease. This suggests that the process of aging may introduce greater risks for canine oral disease.

## Introduction

Microbiomes are the collection of all microorganisms that are found in an environment which are present in all eukaryotic organisms and live in symbiosis with the host. Different microbiomes can be found in the mouth, respiratory tract, urogenital tract and gastrointestinal tract as well as on the skin (Grzeskowiak et al., 2015; Malard et al., 2021). Besides being involved in metabolism, the microbiome is also deeply connected with the health and diseases of the host. At surfaces that are in contact with the external environment (e.g., skin, oral cavity or intestine), the community of diverse microorganisms prevents the establishment of potentially invasive pathogens. The microbiome is also critical in the development and maintenance of the host’s immune system, which learns to recognize resident microorganisms and initiate inflammatory responses against invaders. Dysbiosis or drastic changes in the microbiome are often associated with diseases (Young, 2017; Deo and Deshmukh, 2019; Malard et al., 2021).

The canine oral microbiome is an enormously complex and diverse community within a host organism. Despite the variety of changes in the environment, the oral microbiome remains relatively stable over time and has coevolved with the host organism (Zaura et al., 2014). Depending on the method of delivery (either vaginal or Cesarean section), most organisms acquire their oral microbiome during birth when the newborn is exposed to the mother’s vaginal or gut flora (Zaura et al., 2014). The healthy canine microbiome is defined by common clades of aerobic bacteria, including species from *Actinomyces*, *Porphyromonas*, and *Campylobacter* (Niemiec et al., 2021).

Significant, microbiome-wide changes occur in canines with oral diseases. Periodontal disease is an inflammatory oral disease commonly seen among canines. Compared to a healthy canine oral microbiome, microbiomes of diseased oral cavities exhibit a shift towards anaerobic bacteria (Davis et al., 2013; Santibanez et al., 2021). The abundance of bacteria of the genus *Porphyromonas* increases more than two-fold in the oral microbiome of dogs with periodontal disease (Santibanez et al., 2021). Species including *Porphyromonas cangingivalis* can regulate the host immune response, exacerbating inflammation. Moreover, they can also contribute to the breakdown of the host gingival epithelium (Santibanez et al., 2021). Such oral diseases have been observed at high rates among more senior canines, with frequent oral dysbiosis being observed (Templeton et al., 2023).

Whole genome sequencing (WGS) has been shown to more accurately detect broader microbiome diversity compared to existing 16S amplicon methods (Lewis et al., 2021). A previous study on the metagenomes of aging dogs was able to effectively use WGS to measure longitudinal alpha diversity changes in senior companion dogs (Templeton et al., 2023). However, canine microbiome changes across age groups are not well characterized. Understanding compositional changes related to physical traits will be important for establishing benchmarks when comparing the microbial flora of healthy and diseased dogs where age, sex and weight may be a relevant variable. Age and weight have both previously been correlated with the progression of periodontal disease in canines (Carreira et al., 2015). However, their oral flora was not examined, which leaves both age and weight as possible confounders in the origin of canine periodontal disease. Understanding this relationship will be important for determining how these factors influence canine oral health and its decline. Given previous findings that both periodontal dysfunction and microbial changes are common among aging dogs, it was hypothesized that aging will have a significant relationship with bacterial species associated with oral disease. A longitudinal analysis was not possible due to sampling limitations, but single-factor level correlations could still be used to perform a cross-sectional analysis. This study uses shotgun WGS to characterize metagenomic differences across dogs of varying ages, weights and sexes.

## Results

### Metagenome composition

**Figure 1:**
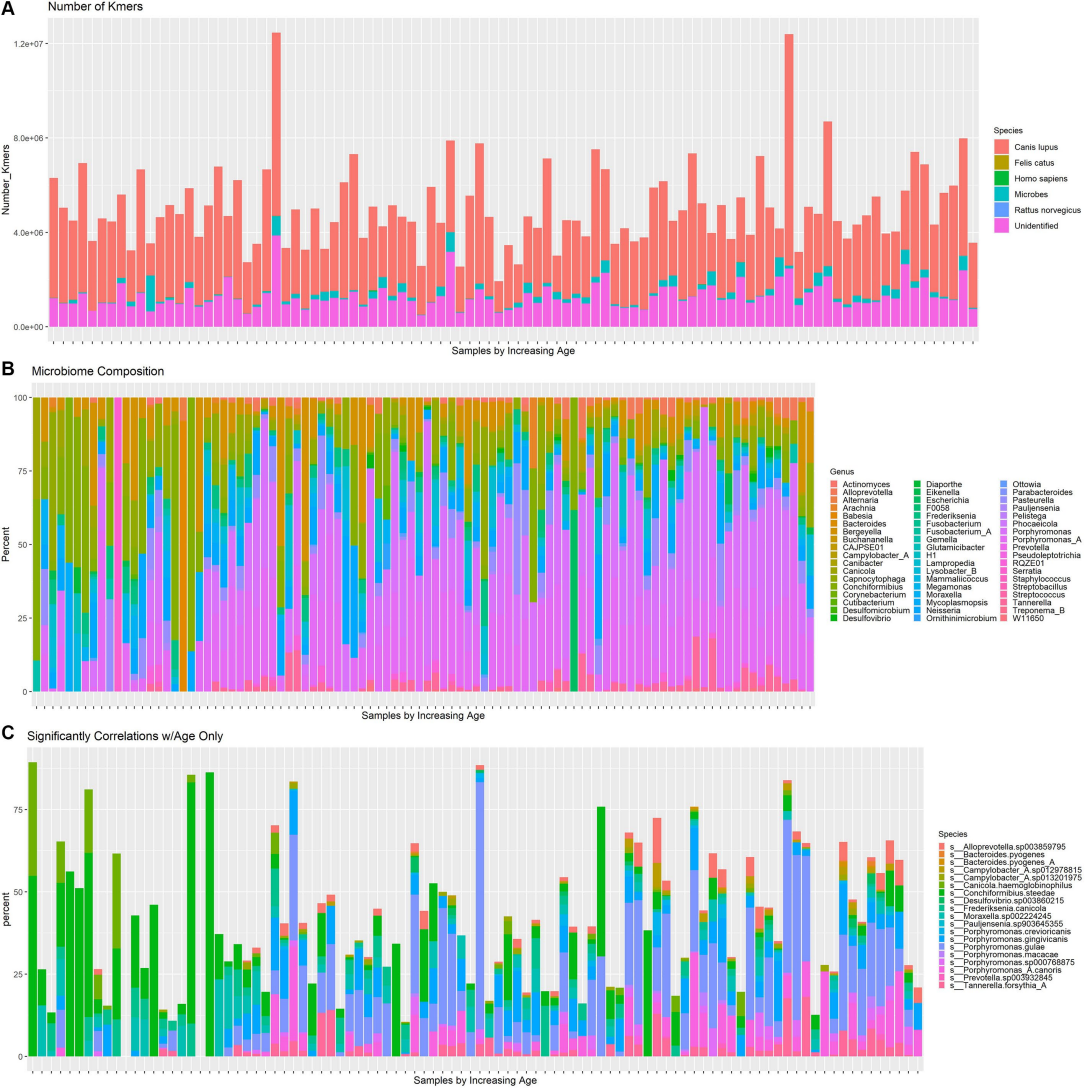
**(A)** Number of kmers corresponding to dog (*Canis lupus*), cat (*Felis catus*), human (*Homo Sapiens*), microbial, brown rat (*Rattus norvegicus*) and unrecognized species in each dog. Samples increase by age left to right. **(B)** Relative abundance of all microbial species with at least 0.01% abundance which shows changes in alpha diversity over time. Samples increase by age left to right. **(C)** Relative abundance of species which have a significant (*p* < 0.05, FDR adjusted) age correlation. Samples increase by age left to right.

Whole genome shotgun sequencing was performed on 96 dogs from the study Rubbi et al. (2022) and profiled using the shotgun taxonomy profiling pipeline on the Aladdin Bioinformatics Platform, which conducted quality assurance and microbiome identification. The kmers species composition is shown in Figure 1A. The majority of kmers belonged to *Canis lupus*. The canine microbiome contained 102 species with more than 0.1% abundance of all kmers belonging to microbial species. The distribution of bacterial abundances across all samples is shown in Figure 1B. Among samples of lower ages, *Conchiformibius* tends to be the dominant genus, while at higher ages *Porphyrmonas* becomes more abundant. The *Moraxella* clade becomes more common in middle age but is less abundant among dogs near the extremes of the age spectrum. The change in abundance of species with significant age correlations is shown in Figure 1C. The abundance of *C. steedae* decreases substantially with increasing age and several *Porphyromonas* species increase over time.

### Species correlated with age

**Figure 2:**
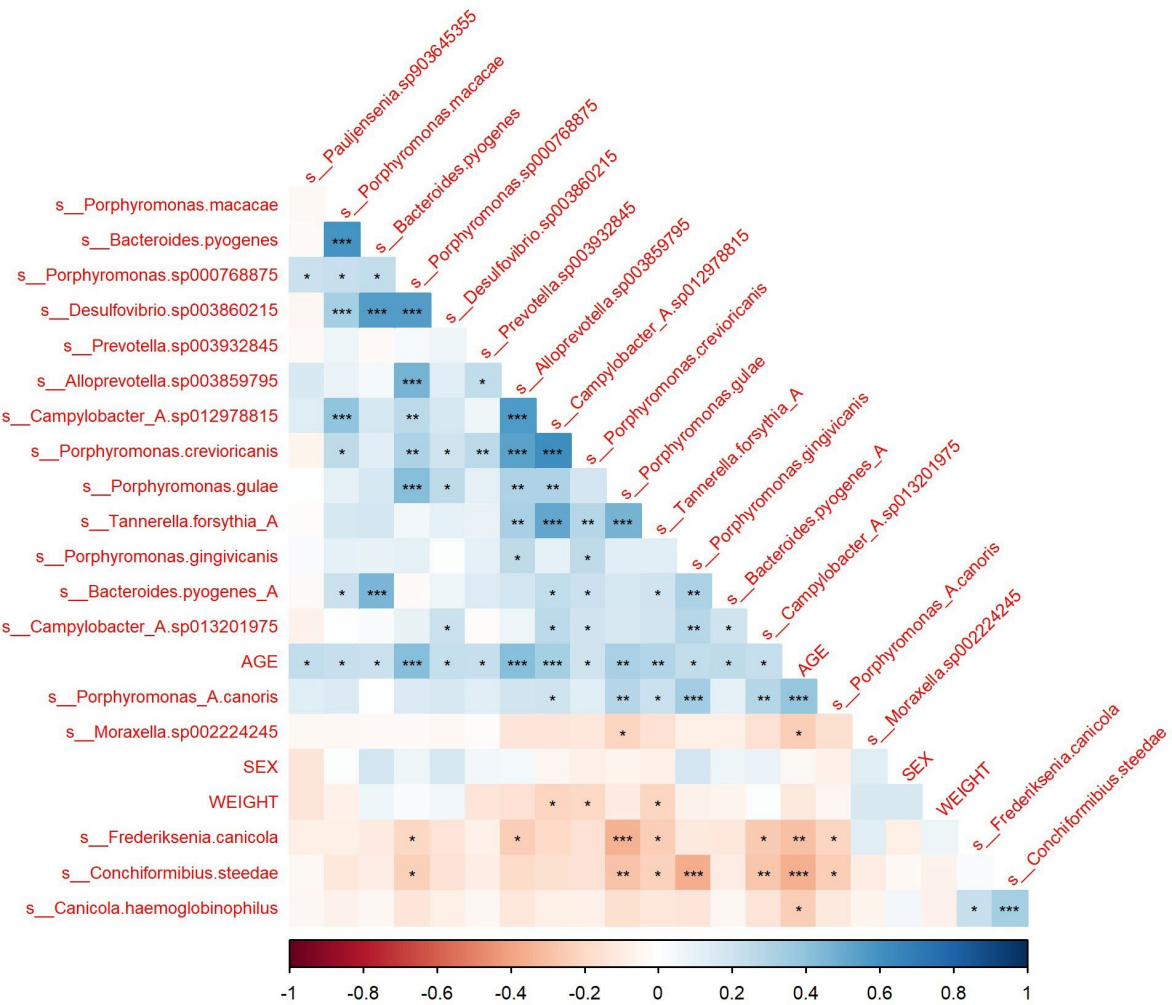
Correlation matrix of species with significant age correlation with sex and weight. *<0.05, **<0.01, ***<0.001.

**Figure 3:**
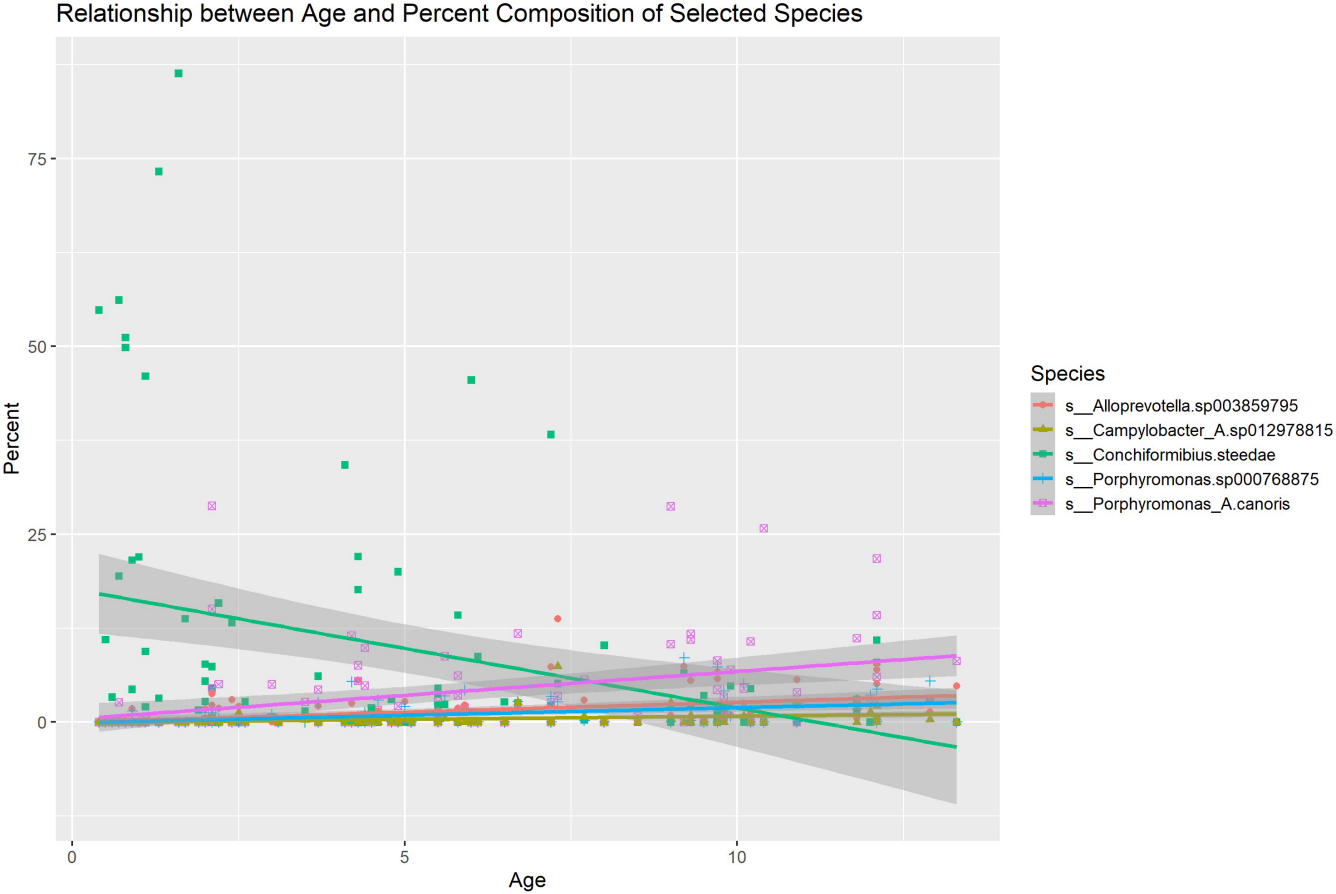
Linear regression describing the change in abundance of species with age correlations with *p* < 0.001.

Pearson correlations between all microbial species, age, sex and weight were computed (Supplementary Figure S1). The correlation matrix of microbial species that exhibit a significant correlation with age is shown in Figure 2. The correlation of microbiome composition with weight and sex is also included in Figure 2. Of the 102 species detected, 19 of them were found to be significantly correlated with age (*p* < 0.05, FDR adjusted). Supplementary Table S1 displays the species’ abundances, age and Simpson’s Index (calculated with the whole microbiome) of each sample. A significant correlation (*p* = 0.0013) was found between Simpson’s Index and age with a coefficient of 0.014016 (standard error: 0.004228), indicating that alpha diversity increased with age. Of the 19 species found to be significantly correlated with age, six belonged to the genus *Porphyromonas*, which all displayed positive Pearson correlations. Four species (*M. sp002224245*, *F. canicola*, *C. steedae*, and *C. haemoglobinophilus*) had negative correlations with age. The change in abundance of species that had significant age correlations with *p* < 0.001 and age is shown in Figure 3. Notably, *C. steedae* has a significant negative correlation with age and quickly decreases with increasing age. Of the 19 species that showed a correlation with age, none showed a significant correlation with sex and only three showed a significant correlation with weight: *Tannerella forsythia*, *Campylobacter sp012978815*, and *Porphyromonas crevioricanis.*

### Canine microbiome samples and microbial species cluster by age group

**Figure 4:**
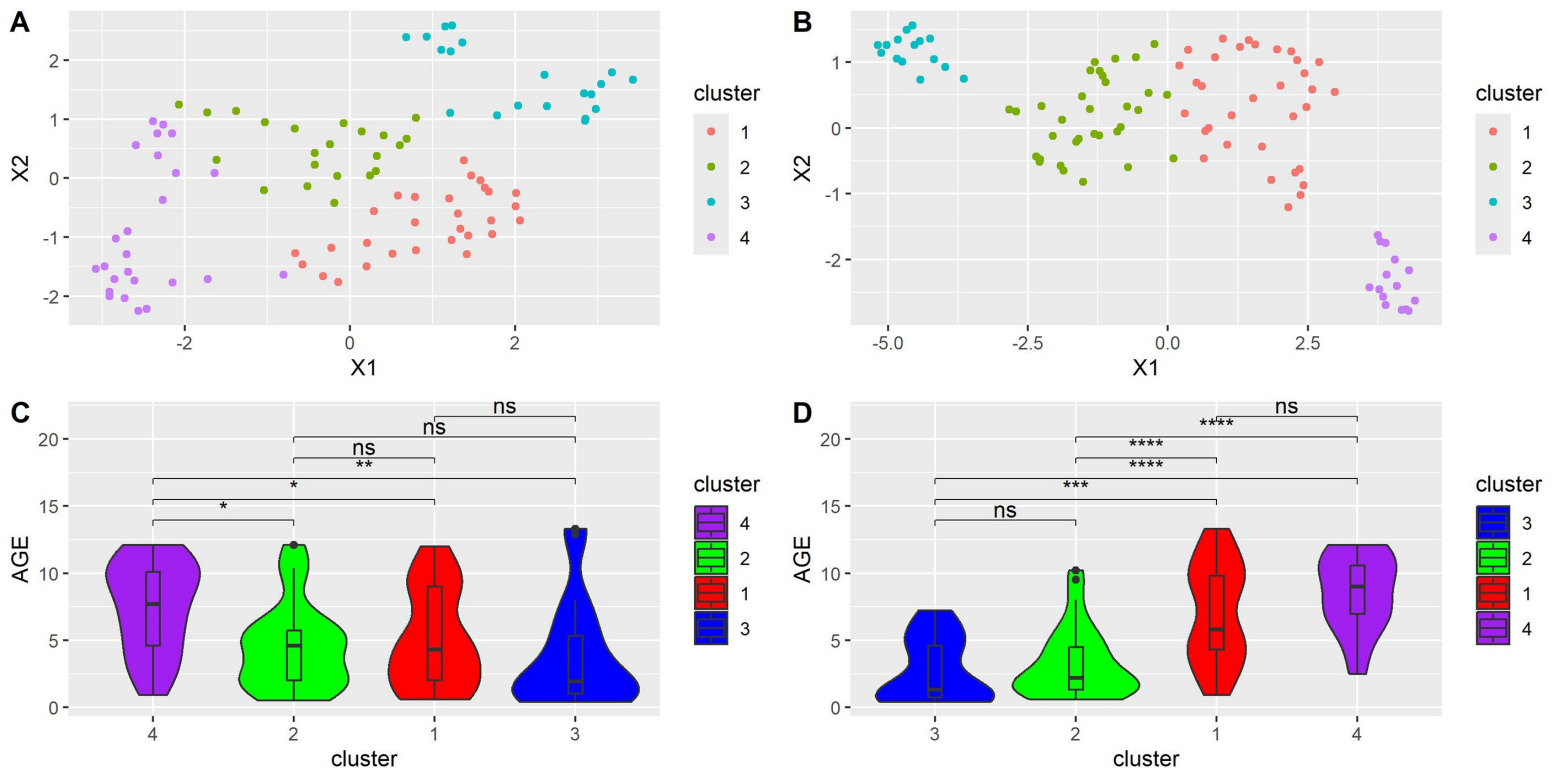
**(A)** UMAP dimension reduction on all species. Each point represents a sample. **(B)** UMAP dimension reduction on species with significant age correlation. Each point represents a sample. **(C)** Violin plots showing differences in age distribution across clusters for **(A)**. Wilcoxon test, FDR adjusted. *<0.05, **<0.01, ***<0.001. **(D)** Violin plot showing differences in age distribution across clusters for **(B)**. Wilcoxon test, FDR adjusted. *<0.05, **<0.01, ***<0.001.

To visualize the relationships between samples in a lower dimensional space, uniform manifold approximation and projection (UMAP) was performed using cosine distance. UMAP analysis was performed twice, once with all species considered and again with only species with significant age correlations. Afterward, k-means clustering was performed to identify groups of similar samples. Silhouette scores were used to find the optimal number of clusters. Although seven clusters were found to be optimal in the UMAP of all samples, the number was reduced to four to remain consistent with the UMAP of only species with significant age correlations. The UMAP and age distributions of all samples when considering all microbial species are shown in Figures 4A,C and of samples when considering only significantly age-correlated species are in Figures 4B,D. Wilcoxon tests were performed to compare age distributions. When all species were considered, Cluster 4 was found to have a significantly different age distribution than Clusters 1, 2, and 3. Clusters 1, 2, and 3 were not found to have significantly different ages from each other. When considering only significantly age-correlated species, Cluster 1 was found to be significantly different from Clusters 2 and 3, but not Cluster 4. Cluster 4 was found to have a significantly different age distribution than Clusters 2 and 3. Cluster 2 was not significantly different from Cluster 3.

**Figure 5:**
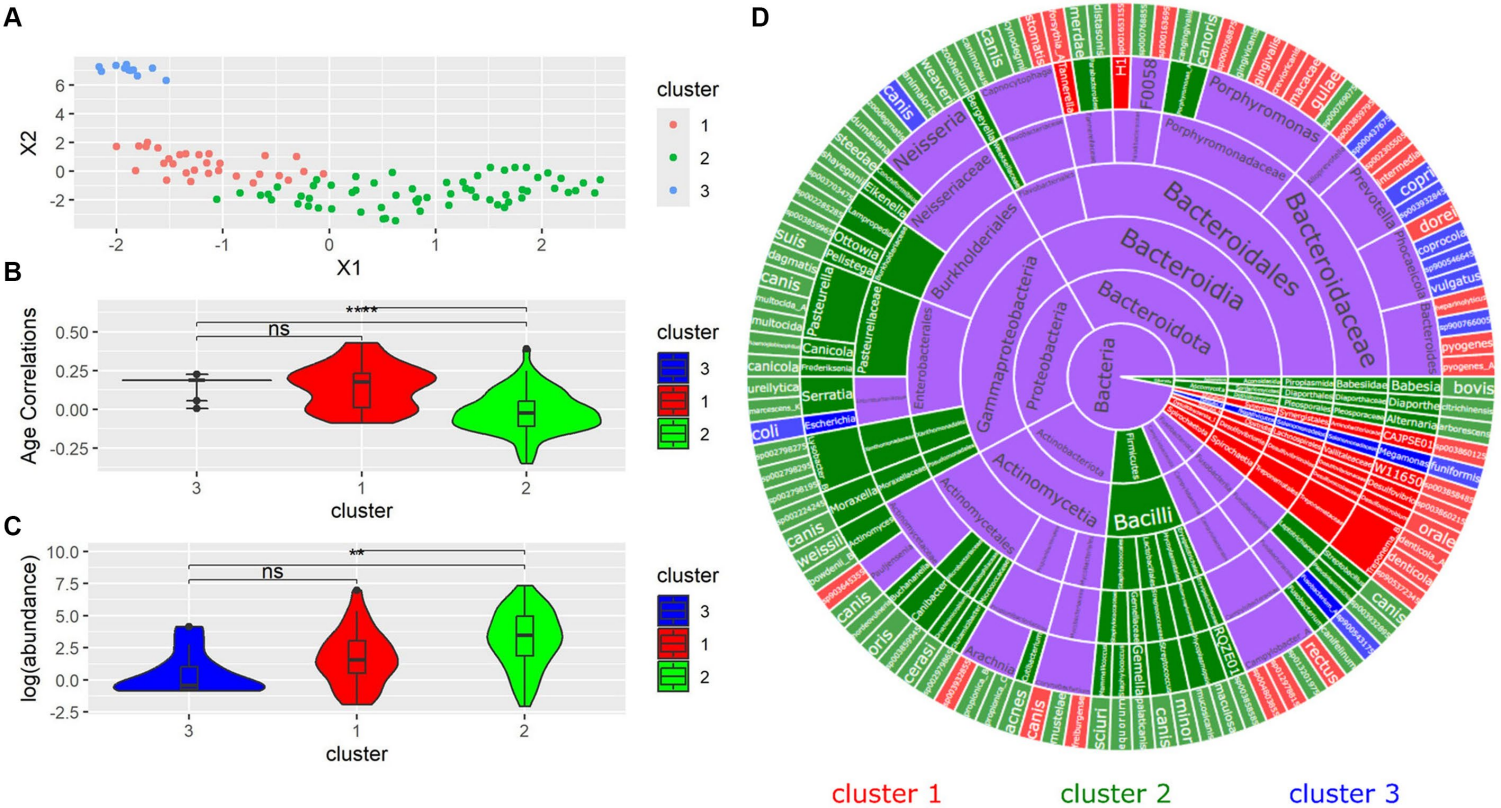
**(A)** UMAP dimension reduction on all samples. Each point represents a species. **(B)** Differences in distribution of age correlations for each cluster. **(C)** Log(abundances) across each cluster. **(D)** Sunburst plot of all microbial species separated by cluster.

Next, the similarity between bacterial species was investigated. Although the Silhouette score indicated that two clusters were optimal, three were used to better resolve different groups of species. From the species UMAP, we identified three clusters (Figure 5A). Clusters 1 and 3 had higher age correlations than Cluster 2, while Cluster 1 and 3 were not shown to have significantly different age correlations between each other (Figure 5B). The distribution of abundances showed a similar pattern where the species in Cluster 2 had a significantly different abundance from those in Clusters 1 and 3. However, the species in Clusters 1 and 3 did not have significantly different abundances (Figure 5C). We sought to investigate the phylogenetic distribution of bacterial species in each cluster. The sunburst plot displays the taxonomy of all species colored by cluster (Figure 5D). Notably, the family Bacteroidaceae contained most Cluster 3 bacteria. Cluster 1 contained most *Porphyromonas* species in addition to all of the detected *Desulfomicrobium*, *Desulfovibrio*, and *Treponema_B* species. Phylum Firmicutes was contained entirely in Cluster 2.

### Age prediction based on microbiome

**Figure 6:**
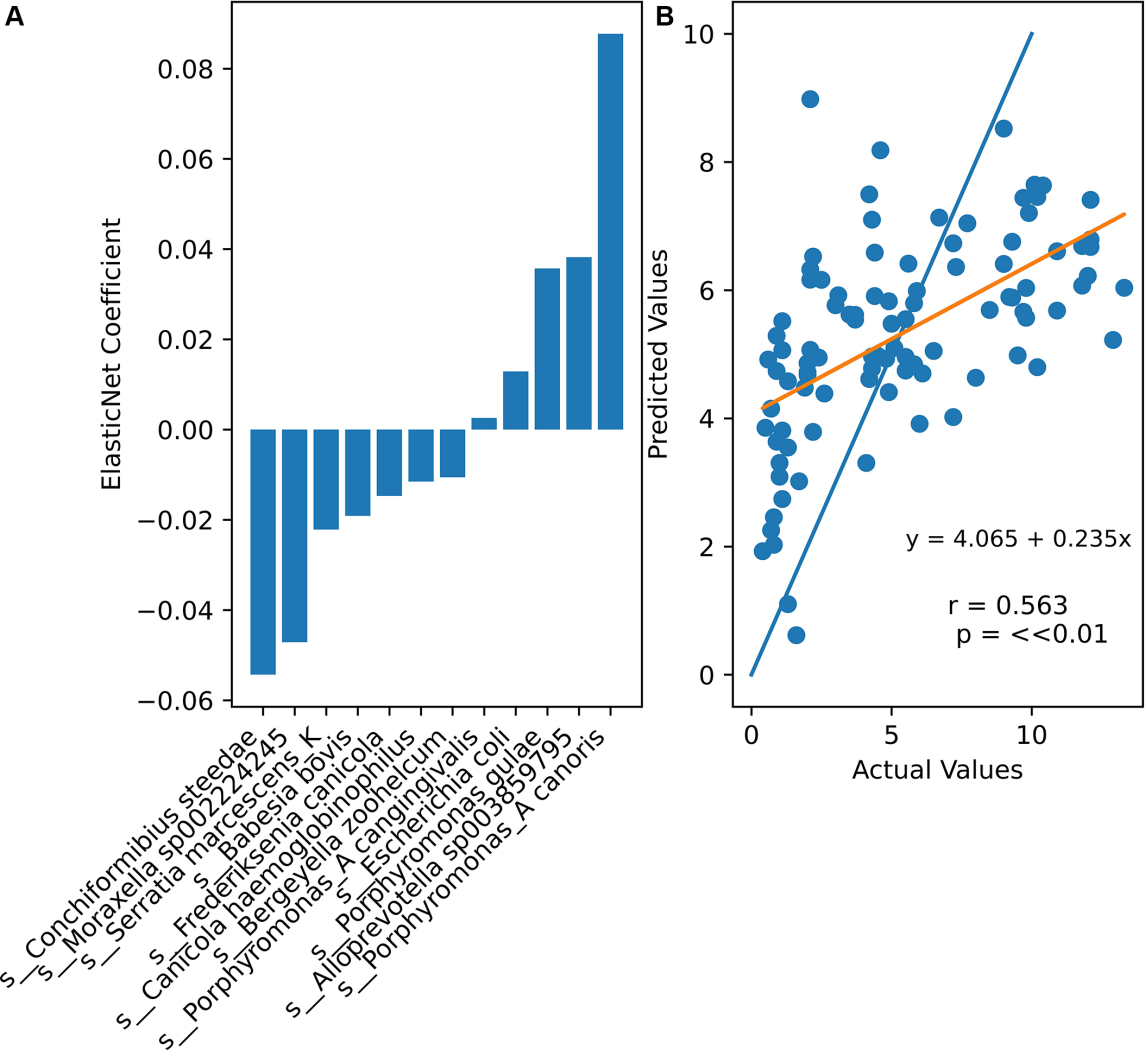
**(A)** Non-zero ElasticNet regression coefficients (alpha = 10, l1 ratio = 0.2). **(B)** Comparison between actual sample ages and ElasticNet predicted ages. Orange line represents the least squares regression line through all points. Blue line is ideal actual = predicted age.

To investigate whether the microbial composition of a canine could be used to predict its age, ElasticNet regression was performed using the abundance of microbial species as features and the age of the dog as the response. Twenty-fold grid cross-validation was performed to find optimal hyperparameters. This cross-validation method tests a range of alpha (orders of magnitude between 1 × 10^−5^ and 100) and l1 ratio (0.2, 0.4, 0.6, or 0.8) combinations to select the ones with the highest accuracy: an alpha of 10 and an l1 ratio of 0.2 were chosen. The results of this cross-validation method are in Supplementary Table S2. Fourteen species had non-zero coefficients (Figure 6). Although a significant correlation was found between the actual and predicted ages, higher real ages were often underestimated, while lower real ages were often overestimated by ElasticNet. The mean squared error of the model was 9.77, meaning that the model tended to predict within ± 3.13 years of the dogs’ actual ages.

## Discussion

Simpson’s Index was found to be positively correlated with age. This is congruent with findings in Templeton et al. (2023), which observed significant increases in bacterial alpha diversity. In human subjects, alpha diversity, as measured by the Shannon index, has been observed to be significantly higher among those with chronic gingivitis and Stage I periodontitis (Kharitonova et al., 2021). This may suggest that the canine’s ability to regulate its oral flora is diminished with age. This may also support that the microbiomes of aging canines resemble those who have periodontal disease. We found that the genus *Porphyromonas* shows a significant positive correlation with age. Species of the genus *Porphyromonas* have been previously observed to be enriched in the oral microbiome of dogs with periodontal disease (Santibanez et al., 2021). *P. gulae* and *P. gingivalis* are both associated with the progression of the disease (Hajishengallis and Lambris, 2012; Nomura et al., 2020). *P. gulae* has been established as part of the core oral bacteriome of dogs with periodontal disease (Niemiec et al., 2021). As an opportunistic pathogen, *P. gingivalis* is capable of subverting a host’s innate immune response and even remodeling the periodontal microbiota (Hajishengallis and Lambris, 2012). It has been observed that in small dog breeds, *P. gulae* likely causes oral disease through the formation of FimA proteins (Yasuda et al., 2024). These proteins polymerize to form fimbriae, which greatly increase *P. gulae* virulence and allow for more rapid formation of biofilms in the gingival margins. This leads to inflammation and eventual tooth decay and loss (Yasuda et al., 2024). The pathogenicity of *P. gingivalis* is largely due to gingipains, which cleave T-cell receptors, immunoglobulins, as well as extracellular matrix components (Bostanci and Belibasakis, 2012). This allows *P. gingivalis* to evade immune response and accumulate to form a biofilm, which leads to periodontitis and dental decay.

By contrast, species from several phyla show negative correlations with age (Santibanez et al., 2021). A prior study compared the relative abundances of various microbial species between the oral microbiomes of healthy dogs and dogs with mild periodontitis through 16S rRNA gene sequencing. In their findings, reductions in *C. steedae* were found to be associated with both gingivitis and periodontitis. Similarly, *C. steedae* abundance had a significant negative correlation with age, suggesting that older dogs have microbiomes more similar to dogs with oral disease. Thus, the oral microbiome of older dogs resembles the periodontal microbiome. Overall, the significant difference in relative abundances of several microbial species between younger and older dogs may be explained by the fact that periodontal disease is more prevalent in older dogs (Nomura et al., 2020). It is also possible that the observed results are a product of natural shifts in oral microbiomes as a result of age. Previous literature has suggested that there may be a genetic predisposition to oral disease and that some breeds may lack resistance to potentially pathogenic strains of bacteria (Wallis and Holcombe, 2020; Wallis et al., 2021). Differences in the longevity of different breeds may also play a role, as those with longer lifespans may accumulate additional subgingival plaque and be at higher risk for periodontal disease. Additional investigation is needed to understand potential genetic risk factors for oral dysbiosis. Understanding potential genetic risk factors or differences in oral dysbiosis among different breeds could help to guide future efforts to prevent the development of canine periodontal disease as veterinarians would be better able to recommend prophylactic action to dog owners. Because samples were collected without medical examination, the disease state of the canines cannot be confirmed. Future research may seek to confirm this possible relationship found in this study by conducting regular health checks in a longitudinal design.

The use of k-means clustering showed that taxonomically unrelated species could be grouped based on similarities in age correlations. Clustering was able to distinguish between clades traditionally associated with canine periodontal disease. Cluster 1 largely contained *Treponema*, *Desulfomicrobium* and many *Porphyromonas* species, which are associated with canine periodontal disease (Harvey, 1998; Riggio et al., 2011). Cluster 3 was characterized by *Prevotella* species, which are similarly implicated in canine periodontal disease and are frequently found in canine plaque and periodontal pockets (Stephan et al., 2008). *N. canis* was also found in Cluster 3, which has been found in canine mandibular abscesses (Cantas et al., 2011). Both *Porphyromonas* and *Prevotella* are associated with human periodontal disease (Stephan et al., 2008). *Conchiformibius* and *Actinomyces*, found in Cluster 2, have been associated with oral health, and declining abundance with worsening oral health (Watanabe et al., 2023). Clusters 1 and 3 both possessed significantly higher age correlations than Cluster 2, suggesting that k-means clustering is able to distinguish between groups associated with both age and disease.

Various approaches have been used to predict human ages using gut microbiome data (Seo et al., 2023). However, there has been no concerted effort to predict canine ages based on their microbiomes. This study was able to predict dogs’ actual ages within 3.13 years of their actual age by using ElasticNet regression, a regularized model. The largest negative coefficient in the ElasticNet model belonged to *Conchiformibius steedae*, which further supports the conclusion that increased age is associated with the decline of beneficial oral flora. Three *Porphyrmonas* species possessed positive coefficients, suggesting that their abundances are positively associated with age. *Porphyromonas* has also been associated with increased periodontal disease severity (Watanabe et al., 2023). While still nascent, being able to predict age and health status based on microbiome composition may be useful for identifying risk factors and deviations from normal aging and canine health. Future studies may look into improving the accuracy of predictive methods and comparing machine learning and regression models, as well as performing longitudinal analyses to better control for microbial changes over time and their relationship to disease progression.

## Methods

### Whole genome sequencing and processing

DNA was extracted from the buccal swabs using the vendor-supplied protocol. Buccal swabs were incubated overnight at 50 degrees Celsius before DNA Extraction. 100 ng of extracted DNA was used for Whole Genome Sequencing (WGS) library preparation. Fragmented DNA was subject to end repair, dA-tailing and adapter ligation using the NEBNext Ultra II Library prep kit using dual unique index adapters (IDT). Libraries were subject to PCR amplification using KAPA HiFi Uracil+(Roche) with the following conditions: 2 min at 98°C; 14 cycles of (98°C for 20 s; 60°C for 30 s; 72°C for 30 s); 72°C for 5 min; hold at 4°C. Library QC was performed using the High-Sensitivity D1000 Assay on a 2200 Agilent TapeStation. Pools of 96 libraries were sequenced on a NovaSeq X Plus (10b lane) as paired-end 150 bases (Rubbi et al., 2022). The mean number of reads was 3,612,534 per canine. The Aladdin Bioinformatics Shotgun Platform, which uses the qiime2 reference databases, was used to process the sequence data, profile taxa and conduct quality assurance (Ewels et al., 2016; Chen et al., 2018; Bolyen et al., 2019). Aladdin uses FastQC to conduct quality assurance by measuring the frequency of duplicated reads as well as G-C content and removing sequences of extreme length (Chen et al., 2018). Sourmash identifies the kmers composition of the samples by comparing kmers to the sourmash-zymo database (Ewels et al., 2016). Sourmash performs taxonomic profiling by creating the smallest possible list of matches to its reference database based on existing k-mers, which are profiled. This smallest possible metagenome is compiled using the method described in Irber et al. (2022), in which containment of a sample hash within the larger reference is calculated using the smallest possible elements from the sample. After finding the match in the reference genome with the highest containment, the match is removed from the sample’s query and the process is repeated. Abundances are estimated using the Jaccard containment of the matched genome within the whole sample metagenome. Qiime2 was used to visualize the composition barplot (Bolyen et al., 2019).

### Correlation and UMAP analysis

The generation of correlation matrices, violin plots, bar plots and scatter plots was done in RStudio version 2023.6.0.421 using the corrr and ggplot2 packages (Wickham, 2016; Kuhn et al., 2022; Posit Team, 2023). The UMAP package was used to perform dimensionality reduction using the procedure described in McInnes et al. (2018). All *p*-values were corrected for using the false discovery rate method for multiple comparisons.

### Age regression model

Jupyter Notebooks were used to generate sunburst plots and the bar and scatter plots for ElasticNet regression using the plotly and sklearn packages (Pedregosa et al., 2011; Plotly Technologies Inc., 2015; Kluyver et al., 2016). Grid Cross Validation tests a range of possible hyperparameters in order to find the optimal settings for ElasticNet regression. For alpha, orders of magnitude between 1 × 10^−5^ and 100 were tested and for l1 ratio the following values were tested: 0.2, 0.4, 0.6, or 0.8. Twenty fold Grid Cross Validation found optimal hyperparameters: l1 ratio = 0.2 and an alpha = 10. The results of all combinations of hyperparameters are available in Supplementary Table S2. These hyperparameters were used by ElasticNet regression to predict dogs’ ages based on their microbiome. A Pearson correlation and associated p-value were computed to measure the effectiveness of the prediction.

## Data availability statement

The datasets presented in this study can be found in online repositories. The names of the repository/repositories and accession number(s) can be found at: https://www.ncbi.nlm.nih.gov/, PRJNA1106914.

## Ethics statement

Ethical approval was not required for the studies involving animals in accordance with the local legislation and institutional requirements because the method of data collection (buccal swab) is non-invasive and did not need ethical approval. Written informed consent was obtained from the owners for the participation of their animals in this study.

## Author contributions

GK: Formal analysis, Investigation, Visualization, Writing – original draft, Writing – review & editing. LZ: Conceptualization, Writing – original draft, Writing – review & editing. LR: Data curation, Writing – original draft, Writing – review & editing. MP: Conceptualization, Data curation, Project administration, Supervision, Writing – original draft, Writing – review & editing.

## Supplementary material

SUPPLEMENTARY FIGURE S1 Correlation matrix of all species, age, weight, and sex.

SUPPLEMENTARY TABLE S1 Age, Simpson’s index of all species in the sample, and relative abundance of species with significant age correlations.

SUPPLEMENTARY TABLE S2 Grid cross validation results.
