# Supplementary figures and images for: Age-correlated changes in the canine oral microbiome
Source: Front Microbiol. 2024 Jul 16;15:1426691. doi: 10.3389/fmicb.2024.1426691 (PMC11287893; doi:10.3389/fmicb.2024.1426691)

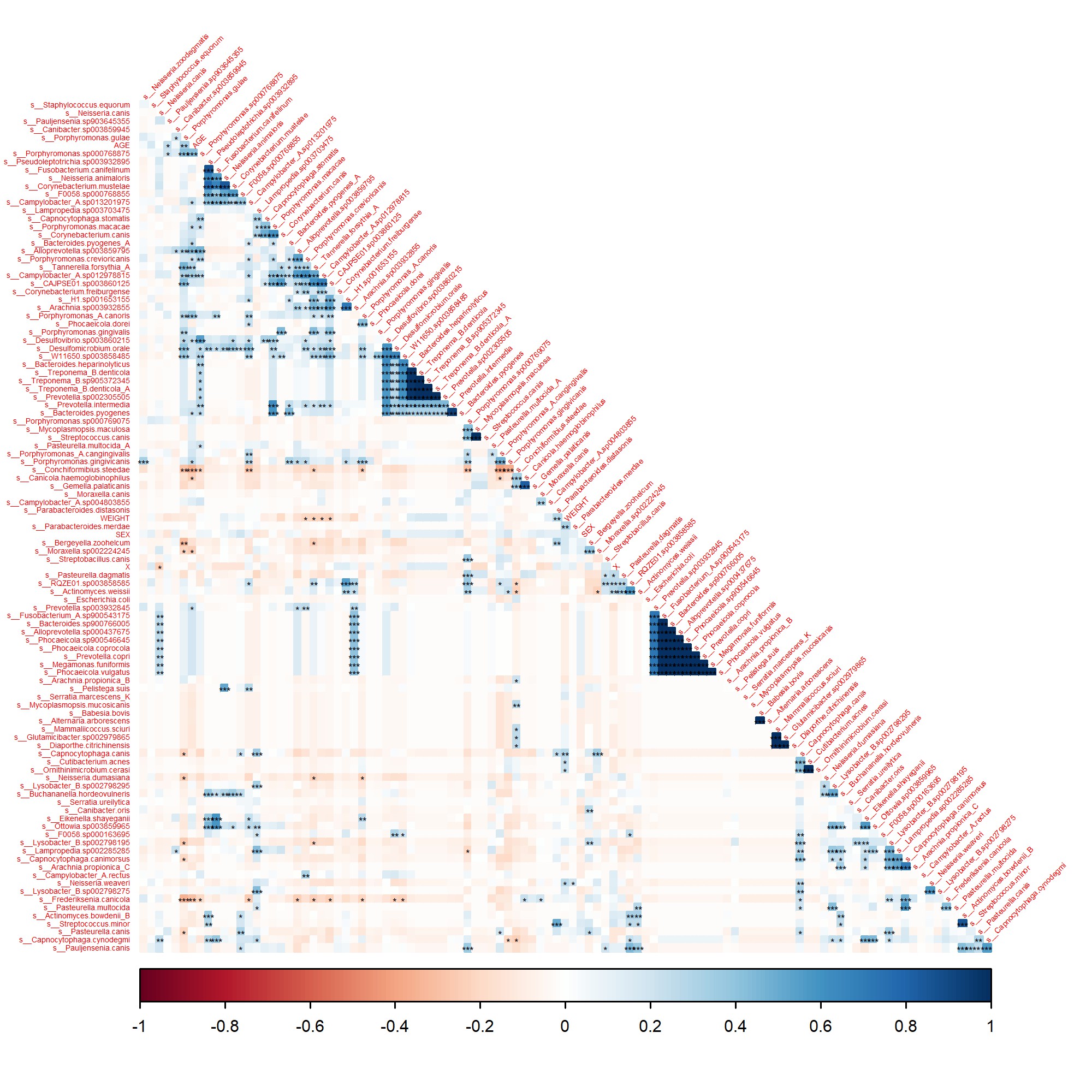

Supplement: SUPPLEMENTARY FIGURE S1 — Correlation matrix of all species, age, weight, and sex. [file Image_1.jpeg]
